# Effects of stair riser height on ankle proprioception in individuals with and without chronic ankle stability

**DOI:** 10.3389/fbioe.2025.1457233

**Published:** 2025-02-07

**Authors:** Xueying Wang, Zheng Wang, Roger Adams, Yang Gao, Jie Lyu, Jia Han

**Affiliations:** ^1^ Department of Sport and Physical Education, Faculty of Arts and Social Sciences, Hong Kong Baptist University, Kowloon Tong, Hong Kong SAR, China; ^2^ School of Exercise and Health, Shanghai University of Sport, Shanghai, China; ^3^ Shanghai Sixth People’s Hospital Affiliated to Shanghai Jiao Tong University School of Medicine, Shanghai, China; ^4^ Research Institute for Sport and Exercise, Faculty of Health, University of Canberra, Canberra, ACT, Australia; ^5^ College of Rehabilitation Sciences, Shanghai University of Medicine and Health Sciences, Shanghai, China; ^6^ Science, Technology, Engineering and Mathematics (STEM) College, RMIT University, Melbourne, VIC, Australia

**Keywords:** chronic ankle instability, motor control, ankle proprioception, stair descent, biomechanics

## Abstract

**Background:**

Ankle sprains during stair descent are prevalent, especially in those with chronic ankle instability (CAI), which may be attributed to diminished ankle proprioception associated with CAI.

**Objective:**

This study aimed to determine whether individuals with CAI have lower ankle proprioceptive performance during stair descent and to determine to what extent stair riser height may affect ankle proprioception.

**Methods:**

40 university students, including 21 CAI (9 males and 12 males, mean age 22.38 years, mean height 169.97 cm and mean weight 64.88 kg) and 19 healthy controls (11 males and 8 males, mean age 23.05 years, mean height 169.42 cm and mean weight 65.18 kg) volunteered. The Ankle Inversion Discrimination Apparatus for Stair Descent (AIDASD) was used to measure ankle inversion proprioception across 3 different riser heights: 15 cm, 17.5 cm, and 20 cm during stair descent.

**Results:**

ANOVA showed that individuals with CAI performed significantly worse than health group across all tested riser heights (F = 44.066, p < 0.001), with a significant main effect of riser height (F = 13.288, p < 0.001). Significant differences in proprioceptive acuity were found between 15 cm and higher risers (*p* < 0.001), but not between 17.5 cm and 20 cm (*p* = 0.675), alongside a significant linear downward trend with increasing riser height (F = 15.476, p < 0.001). No significant interaction was observed between the group and riser height (F = 0.745, *p* = 0.478).

**Conclusion:**

The presence of ankle instability and increased riser height significantly negatively affected ankle inversion proprioceptive performance during stair descent, which may increase the risk of ankle sprain.

**Application:**

Potential applications of this research include the assessment of ankle proprioception during stair descent attributable to effective ankle instability rehabilitation and riser height selection for safe stair design.

## Introduction

Acute lateral ankle sprains are the most common musculoskeletal injuries (Herzog et al., 2019; Wang et al., 2024; Witt and Witt, 2013). In addition, acute ankle sprains have a high rate of recurrence and residual symptoms, which may lead to the development of chronic ankle instability (CAI) (Roos et al., 2017). CAI has been defined as a condition characterized by persistent perceived or episodic giving away and ongoing symptoms such as pain, weakness and reduced self-reported function (Gribble et al., 2016; Hertel and Corbett, 2019).

Over 25% of ankle sprains requiring hospital care over a 4-year period result from a fall downstairs (Waterman et al., 2010). Stair descent is a challenging locomotor task that necessitates complex balance control and involves greater forward acceleration of the upper body compared to level walking (Novak et al., 2016). Furthermore, stair descent places increased demands on the muscle, force, and movement at the ankle joint (Jacobs, 2016). During this process, the ankle joint bears weight and is plantar-flexed, one posture seen in ankle sprains (Wright et al., 2000). In participants with a history of unilateral ankle sprain, participants experiencing increased perceived instability were likely to show greater ankle inversion and plantarflexion during stair descent (Cao et al., 2020). Furthermore, when compared to healthy controls without a history of an ankle sprain, patients with CAI demonstrated excessive tibiotalar inversion during stair descent (Cao et al., 2019).

However, much of the current knowledge about motor control deficits in ankle instability during stair descent is based on biomechanical methods such as kinematics (Cao et al., 2022; Cao et al., 2020; Cao et al., 2019), kinetics, and electromyography (Ghaderi et al., 2021), with fewer studies focusing on the role of proprioception in stair descent, although this plays an essential role in movement control (Henry and Baudry, 2019; Witchalls et al., 2012a).

Proprioception is the capacity to receive sensory input from mechanoreceptors and integrate it with all pertinent sensory information to determine body position and movement, ultimately producing an appropriate motor response (Han et al., 2016). The crucial role of ankle complex proprioception in movement and balance control has been widely acknowledged (Fournier Belley et al., 2016; Han et al., 2015b; Horak and Nashner, 1986). During stair descent, the ankle complex-which includes the ankle joint, the talocrural joint, the subtalar joint, and the inferior tibiofibular joint-is the essential part of the human body in contact with the support surface. Through its distinct mechanoreceptors, ankle joint provides critical sensory information for locomotion regulation in the central nervous system (CNS) (Nurse et al., 2005). Previous research has suggested that individuals with CAI have difficulties in accurately detecting the position of their ankle joint before initial impact for landing, which may increase the likelihood of recurrent ankle sprain (Konradsen and Voigt, 2002). More research on ankle proprioception during stair descent would enable researchers and clinicians to better understand the high risk of ankle sprains, and the pathological alteration after ankle sprains.

To assess the extent of ankle proprioception impairment in CAI during stair descent, we developed a novel apparatus, i.e., the Ankle Inversion Discrimination Apparatus for Stair Descent (AIDASD). This novel apparatus replicates the stair descent task and has demonstrated reliability in evaluating ankle proprioceptive acuity in individuals with and without CAI (Wang et al., 2024). Significant differences were found between the Non-CAI and CAI groups in terms of ankle movement discrimination sensitivity during stair descent.

Improved stair design, including factors such as riser height (the vertical distance from one step to the next), has been stressed as a way to significantly minimize the incidence of stair injuries (Roys, 2001; Tse, 2005). However, much of the knowledge about stair descending deficits in CAI is primarily based on riser height. Increasing riser height has been found to further increase the demands of stair descent by increasing ankle displacements, moments, and powers, along with increased muscle activations, in healthy young adults (Jacobs, 2016; Spanjaard et al., 2008).

Although previous results from studies have shown stair descent deficits in individuals with CAI, no studies have yet examined the impact of different riser heights on ankle proprioception, which may be an essential factor in reducing the incidence of ankle sprain during stair descent.

Our previous studies have shown that in a relaxed, standing, weight-bearing position, the ability to differentiate between different ankle inversion movements worsened as inversion depth increased (Black et al., 2014; Symes et al., 2010). Symes et al. pointed out that this finding is associated with the angles at which the ankle is usually positioned during daily tasks. To be more specific, when the extent of ankle inversion exceeds the range typically encountered during daily activities, there is a notable decline in proprioceptive discrimination of movement at the ankle (Symes et al., 2010). Because individuals may frequently face varying elevations of steps in their everyday experiences and have adapted to diverse step heights, the height of steps may not affect proprioceptive ability. In contrast, there has been an indication of a relationship between the magnitude of displacement towards a goal and error in joint position matching, whereby an increase in the range of movement in the criterion movement is related to a higher degree of error in accurately matching its position in the reproduction movement (Goble, 2010). Goble proposed that individuals employ distance information when replicating joint positions, implying that the error in the results may be influenced by the distance of movement (Goble, 2010). Similarly, our recent studies, using the accuracy of discrimination between movements of different distances made as proprioceptively determined targets, showed worsening discrimination towards the more distant targets when individuals raise the upper limb to overhead targets (Han et al., 2021a), which implies that the acuity of proprioception may decrease with increase in riser height due to the greater movement distance.

Therefore, this study was conducted to investigate the effect of varying riser height on ankle inversion proprioceptive performance in individuals with and without CAI. We hypothesized that, with the riser height of the stairs increasing, the proprioceptive function of individuals both with and without CAI may decrease, with ankle proprioceptive scores being significantly lower in people with CAI.

## Materials and methods

This cross-sectional study was approved by the Committee for Ethics in Human Research at Shanghai University of Sport (approval number: 102772021RT123) and all participants provided written informed consent before data collection commenced.

### Participants

The G*Power software package was used to determine the sample size for 90% power and an expected effect size of 0.25 SD units. It showed that to meet these specifications, there should be at least 18 participants in each group.

In this cross-sectional design study, 40 participants, including 21 with CAI and 19 healthy controls, were recruited via advertisements at Shanghai University of Sport from September 2021 to November 2021. Individuals were classified as having CAI if they had a history of at least 1 significant lateral ankle sprain, a history of at least 2 episodes of ankle joint “giving away” in the 6 months before study enrolment, and a score of ≤24 on the Cumberland Ankle Instability Tool (CAIT). For the healthy control group, the inclusion was no history of ankle injury. Participants were excluded from the study if they had a history of previous surgeries to the lower limb, a history of a fracture in the lower limb, and any lower limb musculoskeletal injury in the last 3 months that interrupted desired physical activity for at least 1 day. Demographic information is reported in Table 1.

**TABLE 1:** Participant demographic information (Mean ± SD).

Characteristic	Group		Difference between groups
	CAI	Non-CAI	
N	21	19	
Gender	M9 F12	M11 F8	
Age (y)	22.38 ± 2.78	23.05 ± 1.43	*t* = 0.973, *p* = 0.338
Height (cm)	169.97 ± 9.79	169.42 ± 8.69	*t* = −0.189, *p* = 0.851
Weight (Kg)	64.88 ± 14.23	65.18 ± 13.14	*t* = 0.070, *p* = 0.945
CAIT score	17.00 ± 3.19	29.10 ± 1.48	*t* = 15.094, *p <* 0.001

SD = standard deviation, CAI = chronic ankle instability, N = number, M/F = male/female, CAIT = cumberland ankle instability tool.

### Instrumentation

The Ankle Inversion Discrimination Apparatus for Stair Descent (AIDASD) tests proprioceptive ability to discriminate between different angles of ankle inversion when participants step onto the wedged platform in a task of stair descent, and its reliability and validity have been verified previously (Wang et al., 2024). The AIDASD apparatus consists of three steps to ensure a full stair gait cycle, the last of which having a wedged landing platform for the testing foot. For each trial, participants were instructed to stand upright on the top of the staircase and to descend stairs at a self-selected speed. The 4 different wedged platforms could provide 4 possible ankle inversion test angles (10°, 12°, 14° and 16°).

In this study, we aimed to identify the influence of riser height on ankle proprioception performance in individuals with and without CAI during stair descent. Standard riser heights in the Australian and Chinese documented public residential stair dimensions were taken into account when selecting the risers employed in this study (15 cm, 17.5 cm, and 20 cm) (Cao et al., 2019; Dundas et al., 2014; Chinese Standard, 2019). The Australian Building Standards AS1657 for stair riser height specify a minimum of 130 mm and a maximum of 255 mm. Therefore, based on the original apparatus of stair geometry, we built three separate custom 3-step wooden staircases without handrails for the testing protocol with different riser heights (X cm × 29 cm × 60 m, height × depth × width, with X = 15 cm, 17.5 cm, and 20 cm).

### Testing protocols

Testing took place in a university clinical laboratory and was conducted by a professional therapist. To standardize the sensory experience from the footplate, all participants were barefoot for AIDASD testing.

**FIGURE 1:**
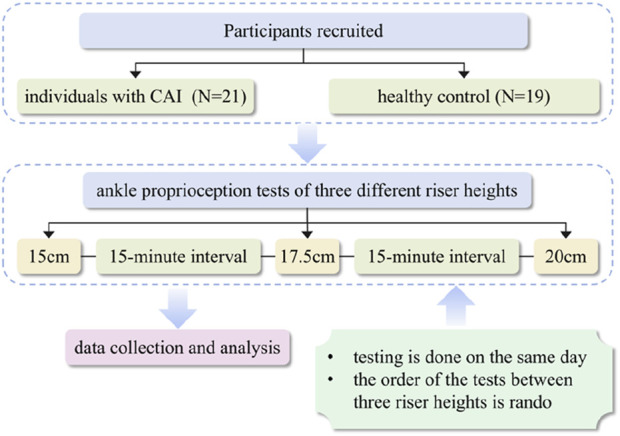
The flowchart of the study.

The data from 3 tests in total on the AIDASD at different riser heights was collected, with a participant completing the test on the same day. To avoid any learning effect, the test sequence was randomised by drawing cards, with tests separated by 15 min of rest (Figure 1).

During the proprioceptive testing process at each riser height, participants were first given a familiarization session in which they experienced the four ankle inversion positions in order three times, going from the smallest (Position 1 = 10°) through to the largest (Position 4 = 16°), for 12 inversion movements in total. After that, the data test was conducted with individuals with CAI who started descending the stairs with their non-symptomatic ankles and control participants starting with the non-dominant lower extremity, with the dominant leg defined as the one they prefer to use when kicking a ball (Xiao et al., 2024). During the process at each riser height, participants were instructed to keep their heads up and eyes directed forwards and not to look down at the apparatus, to eliminate the availability of visual information about the wedged platform. At each riser height participants undertook 40 trials without feedback in total, with 10 randomly presented trials for each different inversion position. On each trial, participants identified the ankle inversion position experienced based on proprioceptive information and responded with a number (1, 2, 3, or 4) reflecting the ankle inversion position they felt that they had just experienced.

### Data analysis

Data were analyzed using SPSS version 24 (IBM Corporation Route 100, Somers, NY 10589). Each participant’s proprioceptive acuity at the ankle complex was assessed by calculating a movement discrimination score. To obtain this acuity score, raw data were initially put into a 4 × 4 matrix indicating the frequency with which each response was made for each stimulus. Three pair-wise receiver operating characteristic (ROC) curves were generated by non-parametric signal detection analysis. Following this, the mean area under the curve (AUC) was obtained using SPSS for each participant, giving an ankle proprioceptive discrimination score that could range from 0.5, equal to chance performance, to 1.0, a perfect score.

A 2-way repeated measures analysis of variance (ANOVA) was conducted using the factors of Group (CAI and non-CAI group) and Riser Height (15 cm, 17.5 cm, 20 cm). After a significant main effect, *post hoc* pairwise comparisons were carried out, with Bonferroni adjustment used to account for multiple comparisons. Effect sizes as partial eta squared (*η* ^*2*^_*p*_) were determined. Effect sizes greater than or equal to 0.25, 0.09, and 0.01 were considered large, moderate, and small, respectively (Cronk, 2019). Polynomial trend analysis was employed to examine the shape of the ankle proprioceptive discrimination sensitivity function across different stair riser heights. A significance level of *p* < 0.05 was adopted for all analyses.

## Results

There was no significant interaction between the Group and Riser Height factors for ankle proprioceptive discrimination sensitivity (F = 0.745, *p* = 0.478, *η* ^*2*^_*p*_ = 0.019). There was a significant group main effect, indicating that the overall AUC proprioceptive discrimination scores in individuals with CAI were significantly lower than in participants without CAI (F = 44.066, p < 0.001 *η* ^*2*^_*p*_ = 0.537). Riser height had a significant main effect on ankle proprioceptive acuity (F = 13.288, p < 0.001 *η* ^*2*^_*p*_ = 0.259). Post hoc pair-wise comparisons revealed a significant difference at 15 cm compared to 17.5 cm (*p* < 0.001, 95% CI = 0.018, 0.045) and 20 cm (*p* < 0.001, 95% CI = 0.021, 0.049), with no significant difference between 17.5 cm and 20 cm (*p* = 0.675, 95% CI = −0.014, 0.021).

**FIGURE 2:**
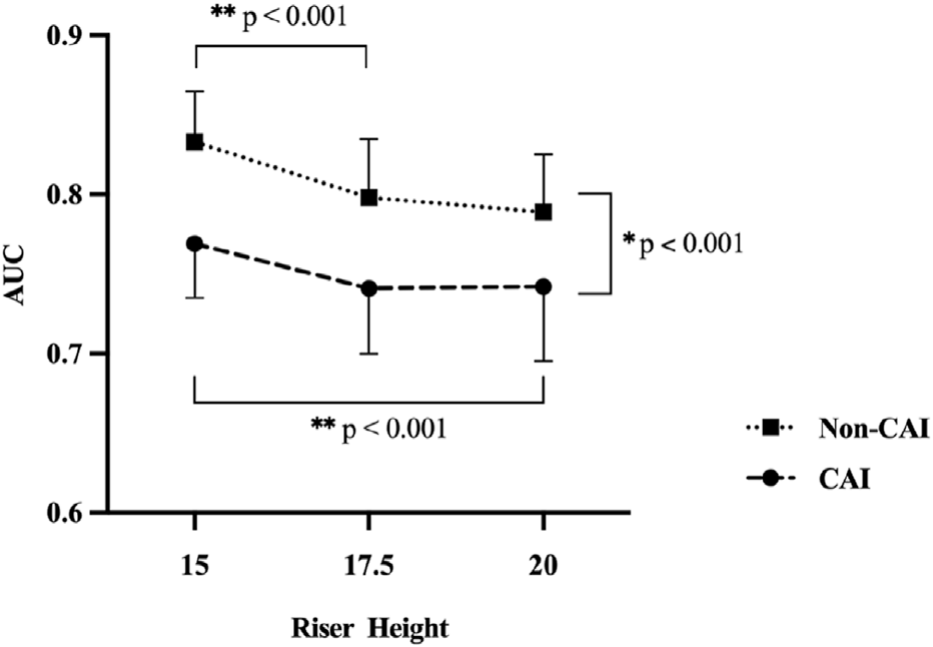
^*^ Indicates a significant difference between groups. ^**^ Indicates a significant difference between riser heights.

The polynomial trend analysis result showed that the linear trend component was significant (F = 15.476, p < 0.001) without a significant quadratic trend (F = 1.770, p = 0.188), indicating that both groups reduced their ankle proprioceptive performance in a similar linear fashion as riser height increased (Figure 2).

## Discussion

This study aimed to examine the impact of changes in riser height on ankle inversion proprioception in individuals with and without CAI. The main effect of riser height observed here showed that riser height can affect ankle proprioceptive ability for both groups, with increased riser height being likely to reduce ankle inversion proprioceptive performance during stair descent. In addition, the group main effect observed here demonstrated that the CAI group had worse ankle proprioceptive performance during stair descent than the non-CAI group. Given the importance of stair descent in daily life and the increased risk of ankle sprain during descending stairs, slight modifications in step geometry can help to reduce the risk. The findings here can be used to inform changes in stair safety codes and standards.

Riser height is identified as a critical element in stairway safety that can help reduce stair injuries (Novak et al., 2016). In this study, results indicate that proprioceptive ability deteriorated as riser height increased, with both groups showing reduced ankle proprioceptive performance in a similar linear pattern as riser height went up. This is consistent with the results observed in the upper limb proprioceptive tasks in a previous study, which reflected worsening discrimination toward the more distant targets (Han et al., 2021a). Previous studies have evaluated the effects of stair riser height on stair descent by using biomechanical methods, with results that show that as riser height increases, the stability of movement reduces, with further demands for the movement system including increasing lower extremity muscle activity (Gerstle et al., 2018) and ground reaction forces (Novak et al., 2016). It has been shown that movement planning is concerned with the specification of feedback gains, and movement control is the use of these feedback gains to driver movement (Gallivan et al., 2018). Among these feedback gains, proprioceptive information may be the more trustworthy source because when vision is employed to track activity in the external environment, the central nervous system may place greater emphasis on proprioceptive information from particular parts of the body for movement control according to the sensory reweighting hypothesis (Han et al., 2015a; Pasma et al., 2012). Therefore, we may speculate that with riser height increasing, the motor system needs to produce a series of changes such as increasing muscle activity to compensate for reduced proprioceptive feedback (Luo et al., 2024).

However, *post hoc* pair-wise comparisons here indicated a significant difference at 15 cm when compared to 17.5 cm and 20 cm, but no significant pairwise difference between 17.5 cm and 20 cm was found. This infers that participants were able to integrate multi-joint proprioceptive inputs more effectively from the hip and knee to help determine the ankle position and movement in space when the riser height was more than 17.5 cm. Dynamic stabilization of joints is accomplished through the integrated functioning of individual joints in the lower limb, collectively creating the lower kinetic chain (Chan et al., 2022). It has previously been shown that CAI patients transitioned the ankle landing strategy from the rearfoot to the forefoot (Gerstle et al., 2017) and increased lower extremity muscle activation (Gerstle et al., 2018) between riser heights of 15 cm and 25 cm, which may prepare for a less stable ankle joint position at landing and a distal foot load. These results suggest that control of the ankle joint deteriorates with riser height increasing and greater hip and knee motions may be required to compensate for maintaining stability. Indeed, kinematic methods have shown that individuals with increased perceived ankle instability are likely to increase hip adduction, hip flexion, knee adduction and knee flexion (Cao et al., 2020). The riser height they used was 18 cm, ranging from 17.5 cm to 20 cm. Therefore, as the riser height increases, irrespective of ankle stability conditions, individuals may change their movement patterns by increasing hip and knee movements to compensate for ankle instability, which leads to more reliance on hip and knee proprioceptive information input. The stair descent task involves a sequence of overlapping joint movements, requiring a complex interplay and coordination among joints (Tripp et al., 2006). It has been shown that proximal joint stability is important for optimal distal function (Tripp et al., 2006). We speculate that this adaptive strategy in CAI with increasing riser height during stair descent may contribute to increasing distal ankle stability and therefore decreasing the risk of re-injury. Thus, further studies could focus on sensory-motor mechanisms of control during stair descent by combining kinesiologic methods and dynamic proprioception tests.

Although the two groups reduced their ankle proprioceptive performance in a similar linear pattern as riser height increased, results also showed a significant group main effect, meaning that, overall, individuals with CAI performed worse than those without CAI in ankle inversion proprioceptive tests during stair descent, regardless of riser height. This is consistent with our previous research, where the AIDASD assessment was found to be reliable and capable of discriminating between individuals with and without CAI (Wang et al., 2024). Additionally, previous studies have shown ankle proprioceptive deficits during dynamic and functional tasks such as stepping, and landing (Han et al., 2021b; Witchalls et al., 2012b). However, a systematic review found no significant difference between individuals with and without CAI when using proprioception assessments, including JPS and TTPDM (Hiller et al., 2011). These conflicting results indicate that JPS and TTPDM may not be sufficiently sensitive to detect the proprioceptive deficits of CAI. Research has demonstrated that ankle proprioception in individuals with CAI is specific to certain tasks and should be assessed while imitating the conditions of an ankle injury (Han et al., 2022; Thompson et al., 2018). Being the same as the Ankle Inversion Discrimination Apparatus for Landing (AIDAL) (Han et al., 2021b), the AIDASD test involves complete weight-bearing, voluntary active movement, and testing by replicating the ankle sprain “action at injury” with a dynamic and functional movement. In addition, different tasks are used to measure ankle proprioception (Han et al., 2016), but their outcomes often are not associated (Yang et al., 2020), suggesting that different tests may target different neurophysiological characteristics of proprioception (Han et al., 2020). Han and his colleague pointed out the distinction between “imposed” and “obtained” proprioception, which might explain why AIDASD is sensitive enough to detect proprioceptive deficits associated with CAI (Han et al., 2020). Unlike “imposed” proprioception assessed by JPS and TTDPM that focuses on threshold activity in mechanoreceptors, the AIDASD task requires “obtain” proprioception, exposing the central processing impairments in individuals with CAI. The AIDASD test appears to be more specific in recognizing central processing issues and sensitive in detecting proprioceptive impairments seen in normal activities, such as stair descent. Its ability to detect deficits during dynamic tasks indicates a central processing deficit rather than peripheral injury rather than peripheral injury, suggesting that additional studies are needed. Further studies could use brain imaging techniques such as functional near-infrared spectroscopy (fNIRS) to explore changes in neural processing between the CNS and CAI-affected ankles, as observed differences in bilateral prefrontal cortex (PFC) and supplementary motor area (SMA) activations might indicate a compensatory mechanism from proprioceptive disruptions after an initial ankle sprain (Luo et al., 2024).

The current study contains several limitations. First, although the participants in the CAI group met the CAI selection criteria recommended by the International Ankle Consortium, some factors including the level of physical activity were not collected and should be considered in future studies. Second, the results of this study may be generalized only to the young population due to the fact that our participants consisted of university student volunteers. Stairs have been identified as a common source of injurious falls among older adults (Jacobs, 2016; Startzell et al., 2000). Further, ankle proprioceptive acuity falls markedly after 75 years of age (Yang et al., 2019). Therefore, future studies should investigate the ankle proprioception performance during stair descent in older adults. Thirdly, the current study only focuses on riser height. Besides riser height, other stair architecture such as tread width (the horizontal distance between edges of adjacent steps), and handrail availability (Jacobs, 2016; Novak et al., 2016). Further study may explore the effect of other stair design factors on ankle proprioception performance.

From the perspective of practical implications, given the importance of stair descent in participation in the community, this study highlights that consideration should be given to having the smallest riser height possible when designing community and domestic spaces for safety. In addition, for clinicians, targeting assessments and interventions at deficits in ankle proprioception during stair descent may also be important for effective CAI rehabilitation.

## Conclusion

The current study demonstrated a significant impact of ankle instability and stair riser height on ankle proprioceptive performance during stair descent. The findings highlight the underlying proprioceptive mechanisms associated with more frequent ankle injuries during stair descent, as well as the association between riser height and ankle injuries. Given the importance of stair descent in participation in the community, this study highlights that consideration should be given to having the smallest riser height possible when designing community and domestic spaces for safety.

## Data Availability

The raw data supporting the conclusions of this article will be made available by the authors, without undue reservation.
